# Enforced GFI1 expression impedes human and murine leukemic cell growth

**DOI:** 10.1038/s41598-017-15866-9

**Published:** 2017-11-16

**Authors:** Judith M. Hönes, Aniththa Thivakaran, Lacramioara Botezatu, Pradeep Patnana, Symone Vitoriano da Conceição Castro, Yahya S. Al-Matary, Judith Schütte, Karen B. I. Fischer, Lothar Vassen, André Görgens, Ulrich Dührsen, Bernd Giebel, Cyrus Khandanpour

**Affiliations:** 1 Department of Hematology, West German Cancer Center, University Hospital Essen, University Duisburg-Essen, Essen, Germany; 2 Institute for Transfusion Medicine, University Hospital Essen, University Duisburg-Essen, Essen, Germany; 3 CAPES Foundation, Ministry of Education of Brazil, Brasilia, 70040-020 Brazil; 4 Department of Endocrinology, Diabetes and Metabolism, University Hospital Essen, University Duisburg-Essen, Essen, Germany; 5 Department of Laboratory Medicine, Karolinska Institutet, Stockholm, Sweden

## Abstract

The differentiation of haematopoietic cells is regulated by a plethora of so-called transcription factors (TFs). Mutations in genes encoding TFs or graded reduction in their expression levels can induce the development of various malignant diseases such as acute myeloid leukaemia (AML). Growth Factor Independence 1 (GFI1) is a transcriptional repressor with key roles in haematopoiesis, including regulating self-renewal of haematopoietic stem cells (HSCs) as well as myeloid and lymphoid differentiation. Analysis of AML patients and different AML mouse models with reduced *GFI1* gene expression levels revealed a direct link between low GFI1 protein level and accelerated AML development and inferior prognosis. Here, we report that upregulated expression of *GFI1* in several widely used leukemic cell lines inhibits their growth and decreases the ability to generate colonies *in vitro*. Similarly, elevated expression of *GFI1* impedes the *in vitro* expansion of murine pre-leukemic cells. Using a humanized AML model, we demonstrate that upregulation of *GFI1* expression leads to myeloid differentiation morphologically and immunophenotypically, increased level of apoptosis and reduction in number of cKit^+^ cells. These results suggest that increasing GFI1 level in leukemic cells with low *GFI1* expression level could be a therapeutic approach.

## Introduction

Acute myeloid leukaemia (AML) is a disease of the bone marrow (BM) characterised by uncontrolled proliferation and impaired differentiation of haematopoietic progenitor cells^1,2^. As a result, abnormal numbers of myeloid progenitor cells emerge from which leukemic blasts arise. Despite advances in the treatment options, the prognosis of AML patients still remains poor. Transcription factors (TFs) play crucial roles in haematopoietic lineage development^3,4^. Increasing evidence suggests that alteration in the level of TFs could lead to rapid malignant transformation^5,6^. Of the various TFs, Growth Factor Independence 1 (GFI1) is a major regulator of haematopoiesis^7–9^. It regulates the emergence of haematopoietic stem cells (HSCs) in the embryo^10,11^ and preserves HSCs quiescence^12–14^. It directs differentiation of progenitors and more mature haematopoietic cell types^15–23^. Constitutive deletion of murine *Gfi1* compromises HSCs’ “stemness”^12,13^, and resultes in a severe neutropenia accompanied by an accumulation of immature, aberrant monocytic cells both in the BM and peripheral blood (PB)^16,24,25^.

Recently, we have shown that reduced levels of GFI1 in AML patients or in different humanized AML mouse models were associated with an inferior prognosis and an accelerated onset of AML^26^. Therefore, we hypothesize that the differentiation block seen in leukemic blasts could be surmounted by increasing the low *GFI1* level towards normal or high *GFI1* gene expression. Here, we report that the upregulation of *GFI1* expression in leukemic cell lines inhibits the expansion of leukemic cells *in vitro*. *In vivo,* increased expression of *GFI1* in a humanized AML mouse model leads to myeloid differentiation based on immunophenotypical and morphological criteria, increased apoptosis and reduction of cKit^+^ cells, a fraction, which is enriched for leukemic stem cells in MLL-AF9 associated AML^27^.

## Results

### Enforced *GFI1* expression promotes differentiation of normal human haematopoietic stem and progenitor cells (HSPCs)

**Figure 1:**
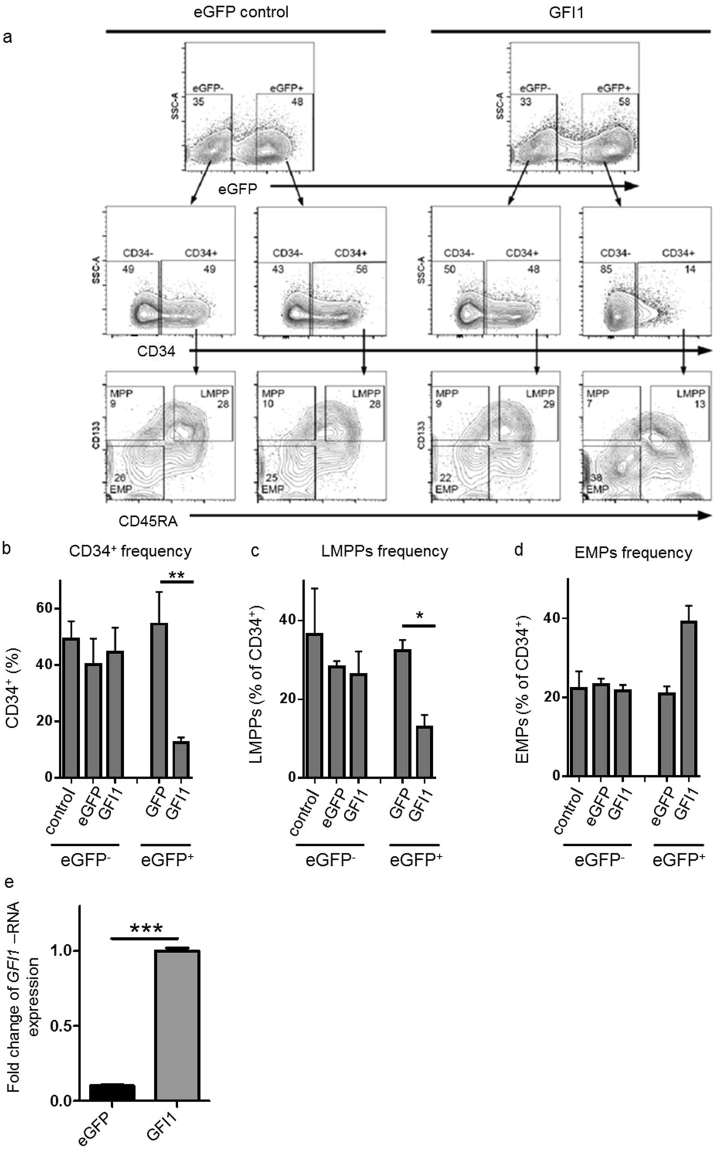
Induced GFI1 expression promotes differentiation of normal human HSPCs. (**a**) Human UCB-derived CD34^+^ cells were transduced with either eGFP (control) or GFI1-eGFP-encoding lentiviral vectors. After 6 days in culture, transduced CD34^+^ cells were analyzed by multi-color flow cytometry to detect various HSPC populations. (**b**) The frequency of CD34^+^ cells gated within the eGFP^-^ as well as the eGFP^+^ fraction of unmanipulated cells (control), eGFP- or GFI1-eGFP-transduced cells is shown (p = 0.048). (**c**) The frequency of lymphoid-primed multipotent progenitors (LMPPs) gated within eGFP^-^ as well as the eGFP^+^ fraction of eGFP- or GFI1-eGFP- transduced cells is shown (n = 3), (p = 0.01). (**d**) The frequency of erythro-myeloid progenitors (EMPs) gated within the eGFP^-^ as well as the eGFP^+^ fraction of eGFP- or GFI1-eGFP- transduced cells is shown (p = 0.07). (**e**) Fold change of GFI1-RNA expression of the eGFP^+^ fraction or GFI1-eGFP- transduced CD34^+^ -cells (p < 0.0001).

To investigate whether increased expression of *GFI1* might impede leukaemia development, we first examined the effect of enforced *GFI1* expression *in vitro* by using human haematopoietic stem and progenitor cells (HSPCs). HSPCs were derived from human umbilical cord blood cells (UCB) obtained from unrelated donors after informed consent according to the Declaration of Helsinki. Human UCB-derived CD34^+^ cells were highly enriched by magnetic cell separation and, subsequently, 5 × 10^4^ CD34^+^ cells were transduced with either an enhanced green fluorescent protein (eGFP) or *GFI1-eGFP*-encoding lentivirus (pCL6-IRES-EGwo backbone) (Supplementary Fig. S1a). Six days after transduction, the various HSPC populations were analysed by multi-colour flow cytometry according to our previous studies^28,29^ (Fig. 1a, Supplementary Fig. S1b). We found that *GFI1* overexpression promoted HSPC commitment into more mature progenitor stages, indicated by reduced percentage of CD34^+^ cells (Fig. 1b) and lymphoid-primed multipotent progenitors (LMPPs) (Fig. 1c) as well as an increase in erythro-myeloid progenitors (EMPs) frequency (Fig. 1d). Cells transduced with *GFI1-eGFP*-encoding lentiviruses showed an 8-fold higher *GFI1*-RNA expression compared to the cells transduced with an *eGFP*-encoding lentivirus (Fig. 1e).

### Overexpression of *GFI1* inhibits *in vitro* expansion of human AML cell lines

**Figure 2:**
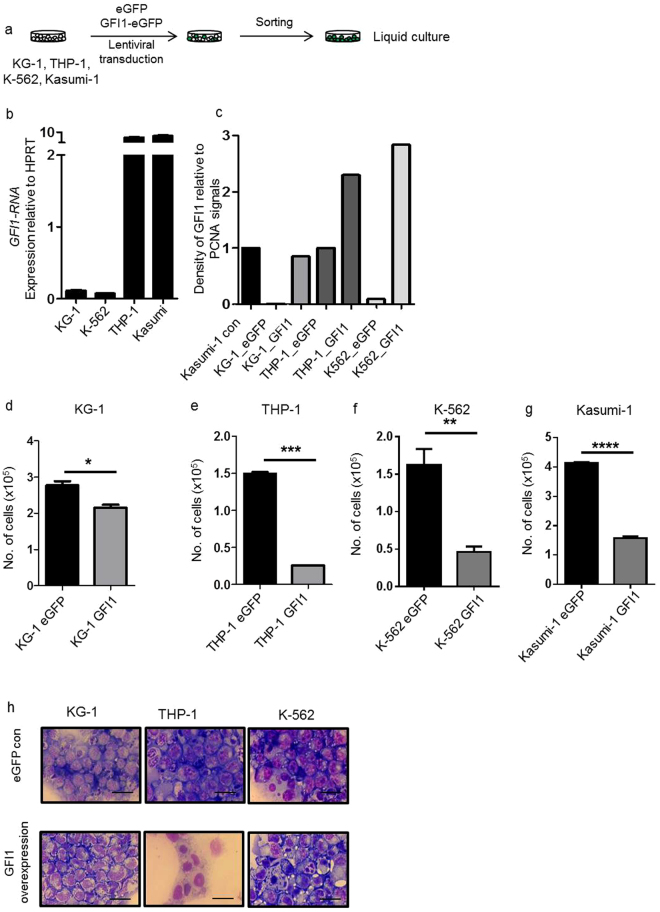
Induced *GFI1* expression inhibits expansion of human AML cell lines in liquid culture. (**a**) Schematic representation of lentiviral transduction of KG-1, THP-1, Kasumi-1 and K-562 cells with *eGFP* control or *GFI1-eGFP* lentiviral vectors. (**b**) Relative *GFI1*-RNA expression of non-transduced KG-1, THP-1, Kasumi-1 and K-562 cells normalized to HPRT-expression (in triplicates). (**c**) Density of GFI1 relative to PCNA. Values of the density from the different GFI1 protein bands relative to the bands of PCNA protein in transduced Kasumi-1, KG-1, THP-1 and K-562 cells either with the *GFI1-eGFP* or *eGFP* control vector are shown here. (**d**) Total numbers of sorted KG-1 cells transduced with the *eGFP* control or the *GFI1-eGFP* after 6 days in liquid culture (n = 2, in triplicates, p = 0.015). (**e**) Total numbers of sorted THP-1 cells transduced with the *eGFP* control or the *GFI1-eGFP* vector after 6 days in liquid culture (n = 2, in triplicates, p = 0.00012). (**f**) Total numbers of sorted K-562 cells transduced with *eGFP* control or the *GFI1-eGFP* vector after 6 days in liquid culture (n = 2, in triplicates, p = 0.002). (**g**) Total numbers of sorted Kasumi-1 cells transduced with the *eGFP* control or GFI1-eGFP vector after 3 days in liquid culture (n = 2, in triplicates, p < 0.00001). (**h**) Cytospins of the sorted KG-1, THP-1, Kasumi-1 and K-562 cells with transduced with the *eGFP* control or *GFI1-eGFP* vector after 6 days in liquid culture. The bar represents 10 µm.

These results led us to examine whether a similar effect of *GFI1* overexpression could be observed in several widely used human leukemic cell lines such as KG-1, THP-1, Kasumi-1 and K-562 (Fig. 2a, Supplementary Fig. S2). Physiologically, these cell lines express different levels of GFI1 (Fig. 2b). By Western Blot analysis we could observe an increase of GFI1 protein levels in the different cell lines after GFI1 upregulation (Fig. 2c, Supplementary Fig. S3a–c). The protein levels in the cell lines with upregulated expression of *GFI1* were at maximum 2-3 times higher than the levels found physiologically in the different cell lines such as Kasumi-1 and THP-1 (Supplementary Fig. S3a,b). To estimate potential impacts on cell proliferation, we cultured these cells in liquid medium for 3 or 6 days, respectively. Increased GFI1 levels led to a significant inhibition of proliferation when compared to cells transduced with only *eGFP*-encoding vectors (Fig. 2d–g). Furthermore, a differentiation of these cells towards granulocytes could be observed (Fig. 2h). For the case of *GFI1*-overexpression in Kasumi-1 cells, we could not prepare a cytospin, since the number of cells was dramatically reduced after *GFI1* overexpression (see below).

**Figure 3:**
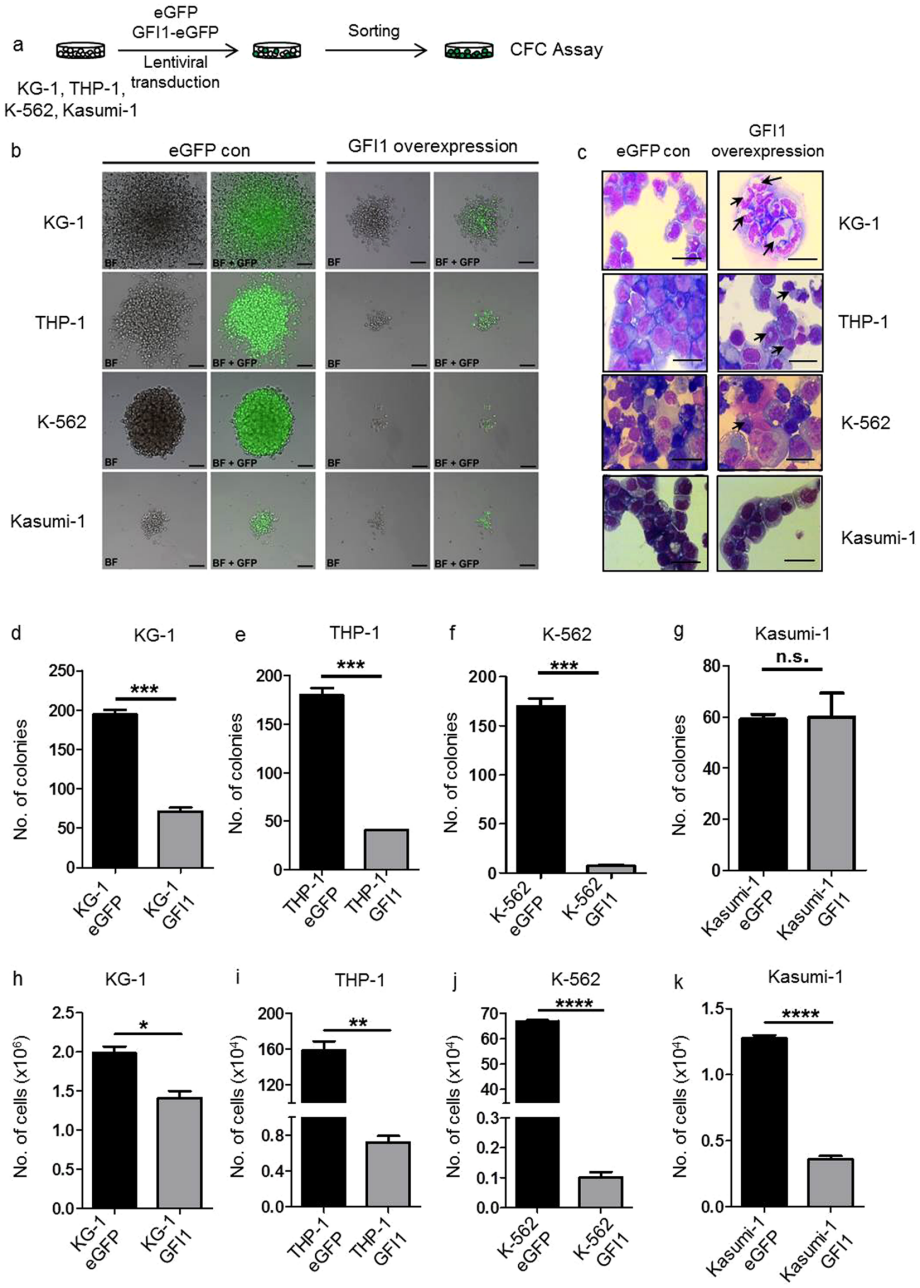
Induced *GFI1* expression reduces CFU capacity and colony formation capability of human AML cell lines. (**a**) Schematic representation of lentiviral transduction of KG-1, THP-1, Kasumi-1 and K-562 cells with the *eGFP* control or the *GFI1-eGFP* lentiviral vectors for CFC assay. (**b**) Examples of colonies derived from KG-1, THP-1, K-562, and Kasumi-1 cells transduced with the *eGFP* control or *GFI1-eGFP* lentiviral vectors after 14 days of culture in semi-solid medium (Methocult GF H4434) (n = 2). The bar represents 50 µm. (**c**) Cytospins of sorted KG-1, THP-1, Kasumi-1 and K-562 cells transduced with the *eGFP* control or *GFI1-eGFP* vectors after 14 days of culture in semi-solid medium (n = 2). The bar represents 10 µm. Differentiated cells are marked with arrows. (**d**) Total numbers of KG-1 colonies derived from cells transduced with the *eGFP* control or the *GFI1-eGFP* lentiviral vectors after 14 days of culture in semi-solid medium (n = 2, p < 0.0001). (**e**) Total numbers of THP-1 colonies derived from cells transduced with the *eGFP* control or *GFI1-eGFP* lentiviral vectors after 14 days of culture in semi-solid medium (n = 2, p < 0.0001). (**f**) Total numbers of K-562 colonies derived from cells transduced with the *eGFP* control or the *GFI1-eGFP* lentiviral vectors after 14 days of culture in semi-solid medium (n = 2, p < 0.0001). (**g**) Total numbers of Kasumi-1 colonies derived from cells transduced with the *eGFP* control or the *GFI1-eGFP* lentiviral vectors after 14 days of culture in semi-solid medium (n = 2, n.s. = not significant). (**h**) Total numbers of KG-1 cells transduced with the *eGFP* control or the *GFI1-eGFP* lentiviral vectors after 14 days of culture in semi-solid medium (n = 2, p = 0.01). (**i**) Total numbers of THP-1 cells transduced with the eGFP control or the GFI1-eGFP lentiviral vectors after 14 days of culture in semi-solid medium (n = 2, p = 0.004). (**j**) Total numbers of K-562 cells transduced with the *eGFP* control or the *GFI1-eGFP* lentiviral vectors after 14 days of culture in semi-solid medium (n = 2, p < 0.00004). (**k**) Total numbers of Kasumi-1 cells transduced with the *eGFP* control or the *GFI1-eGFP* lentiviral vectors after 14 days of culture in semi-solid medium (n = 2, p < 0.00001).

We then plated the transduced cells in semi-solid medium to verify whether *GFI1* overexpression not only influences expansion but also the ability of cells to generate colonies, hence an indication, whether markers of stemness were altered. Lentiviral transduction of *GFI1*-e*GFP* containing vectors into KG-1, THP-1, K-562 and Kasumi-1 human AML cell lines inhibited their clonogenic capacity in semi-solid medium (Fig. 3a) as indicated by smaller colonies and cell numbers compared to control e*GFP*-transduced cells (Fig. 3b,d–k). In addition, enforced *GFI1* expression resulted in the differentiation of these cells, as highlighted by the altered structure of the nuclei and the cytoplasm (Fig. 3c). We also observed, with the exception of Kasumi-1 cells, a reduced number of colonies after *GFI1* overexpression. Kasumi-1 is an AML cell line, which highly expresses *GFI1*, so probably a further increase might have less effect on its ability to form colonies as compared to the other cell lines with lower expression of *GFI1* (Fig. 2b).

### Ectopic expression of *GFI1* impedes the *in vitro* expansion of murine pre-leukemic cells

**Figure 4:**
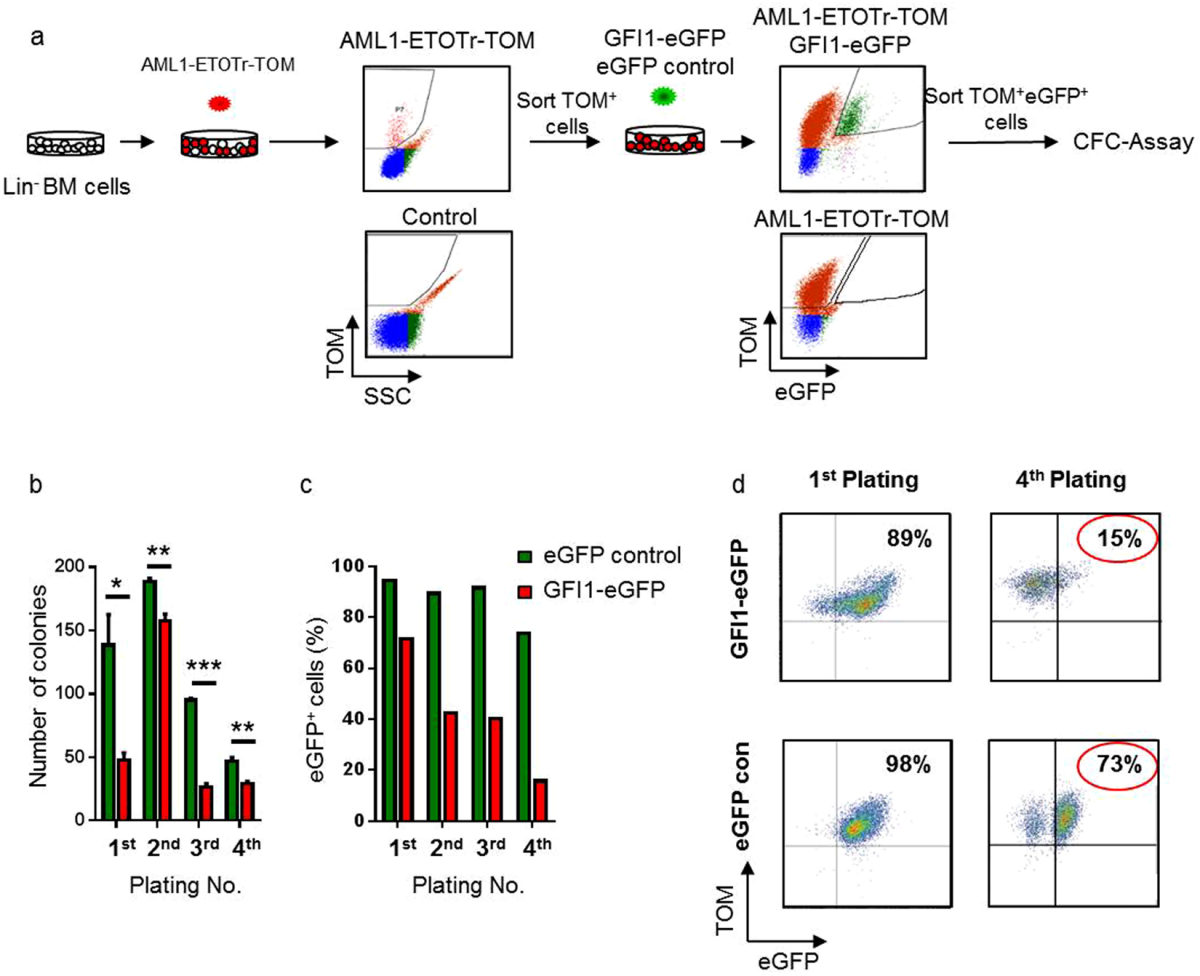
Enforced expression of GFI1 impedes the expansion of murine pre-leukemic cells. (**a**) Schematic illustration of the experimental design. WT murine Lin^-^ BM cells were transduced with AML1-ETOtr-TOM. Sorted cells were subsequently transduced with either *GFI1-eGFP* or *eGFP* control retroviral vectors. After a few days of expansion, double positive cells (*eGFP* ^+^ *TOM* ^+^) were sorted and 1000 cells were seeded in 1 mL MethoCult GF M3434 medium for CFU-C assay. After 6–10 days the number of colonies was counted and 1000 cells/well were serially replated three more times. (**b**) Effects of enforced *GFI1* expression on serial replating of pre-leukemic cells in methylcellulose assays. Values shown are means ( ± SD) (n = 3). The experiment was performed three times in triplicates. (n = 3; *p = 0.021; **p = 0.007; ***p < 0.0001). (**c**) Effects of enforced *GFI1* expression on the percentage of *eGFP* positive cells over serial replating of pre-leukemic cells (n = 3). (**d**) Representative FACS plots of cell analyses from the first and the fourth plating of AML1-ETOtr-TOM Lin- BM cells transduced with either *GFI1-eGFP* or *eGFP* control retroviral vectors.

The above presented data gave a first hint that GFI1 plays a dose-dependent role in leukaemia progression. To further assess the influence of *GFI1* overexpression in primary cells and to test whether the findings can be recapitulated in primary cells, we transduced wild type (WT) lineage negative (Lin^−^) murine BM cells with a retroviral vector encoding the truncated form of the AML1-ETO fusion protein (AML1-ETOtr). This fusion oncoprotein encoded as the result of the t(8;21)(q22;q22) translocation which is frequently found in AML patients, and its expression in murine haematopoietic cells leads to the emergence of an AML similar to the situation found in AML patients^30,31^. Co-expression of *AML1-ETO*tr together with cDNA of the fluorescent reporter gene Tomato (*AML1-ETO*tr-*TOM*) enables detection of the transduced cells. After sorting, the cells were transduced again with either *GFI1*-e*GFP* or e*GFP* control vectors, followed by a second round of sorting after which 1000 eGFP^+^ TOM^+^ cells/well were used for colony forming cell (CFC) assay. The number of colonies was counted after 6–10 days and 1000 cells per well were serially replated another three times (Fig. 4a). The number of colonies was significantly higher in cultures of e*GFP* control-transduced cells than in the *GFI1*-e*GFP* transduced cells (Fig. 4b). We also observed a decrease in the percentage of eGFP^+^ cells in the *GFI1*-e*GFP* overexpressing cells compared to *eGFP*-transduced cells, indicating that *GFI1* overexpressing cells have a competitive disadvantage (Fig. 4c and d). In these experiments overexpression of GFI1 severely impeded the expansion of AML1-ETOtr cells and we were not able to obtain enough material for protein detection. These data confirm our results obtained with human leukemic cell lines (Kasumi-1 also express AML1-ETO) and demonstrate that enforced expression of the *GFI1* gene impedes the *in vitro* expansion and clonogenic capacity of murine pre-leukemic cells.

### Induced *GFI1* upregulation in a humanized *GFI1*-KD AML mouse model promotes morphologically differentiation of leukemic cells

**Figure 5:**
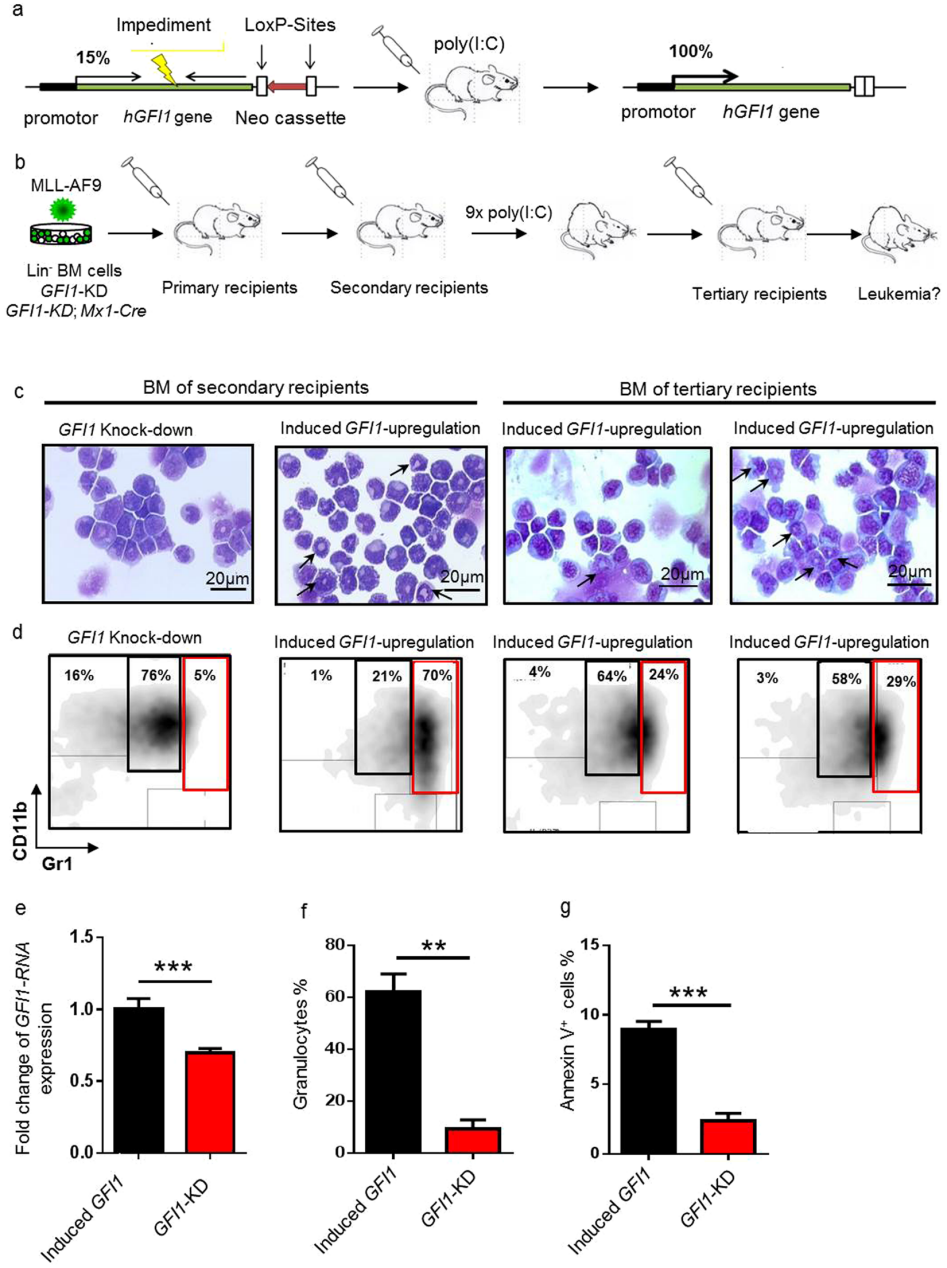
Induced upregulation of *GFI1* leads to morphologically differentiation and increased apoptosis in an AML mouse model. (**a**) Schematic representation of the endogenous murine Gfi1 locus replaced by the human *GFI1* gene in front of a Neomycin (Neo) selection cassette flanked by loxP sites inserted in an antisense direction that results in a reduced expression of *GFI1*. In *GFI1-KD; Mx1-Cre* transgenic mice Cre-mediated excision of the Neo cassette is achieved after administration of poly (I:C), leading to an induced upregulation of the *GFI1* expression level. This does not occur in the *GFI1-KD* mice. (**b**) Schematic illustration of the experimental design. Lin^-^ BM cells derived from *GFI1-KD* or *GFI1-KD; Mx1-Cre* transgenic mice were transduced with a *MLL-AF9*-expressing retrovirus and transplanted in lethally irradiated mice. Approximately 1 × 10^5^ GFP^+^ BM cells of moribund mice were subsequently transplanted into sublethally irradiated secondary recipient mice. Both *GFI1-KD* and *GFI1-KD; Mx1-Cre* transgenic mice were injected with poly (I:C). Mice were observed for signs of disease and sacrificed when moribund. Afterwards 1 × 10^5^ GFP^+^ BM cells of the moribund secondary transplanted mice were transplanted into sublethally irradiated tertiary recipients. (**c**) Different magnifications of Wright-Giemsa staining of BM cytospins derived from mice transplanted with *MLL-AF9 GFI1-KD* or *GFI1-KD; Mx1-Cre* transgenic cells. Bars represent 20 µm respectively. Differentiated cells are marked with arrows. (**d**) Representative FACS plots from the BM of leukemic mice transplanted with *MLL-AF9 GFI1-KD* or *GFI1-KD; Mx1-Cre* transgenic cells from secondary and tertiary recipient mice, showing Gr-1 and CD11b expression gated within the GFP^+^ fraction. The red squares indicate the percentage of granulocytes, while the black squares indicate the percentage of monocytes. (**e**) Fold change of *GFI1*-RNA expression from BM cells of mice secondary transplanted with *MLL-AF9 GFI1-KD* or *GFI1-KD; Mx1-Cre* transgenic cells, (p < 0.01). (**f**) The frequency of granulocytes in the BM of mice transplanted with *MLL-AF9 GFI1-KD* or *GFI1-KD; Mx1-Cre* transgenic cells (n = 4), (p ≤ 0.0021). (**g**) The frequency of Annexin V^+^ cells in the BM of mice transplanted with *MLL-AF9 GFI1-KD* or *GFI1-KD; Mx1-Cre* transgenic cells (n = 4), (p = 0.0002).

**Figure 6:**
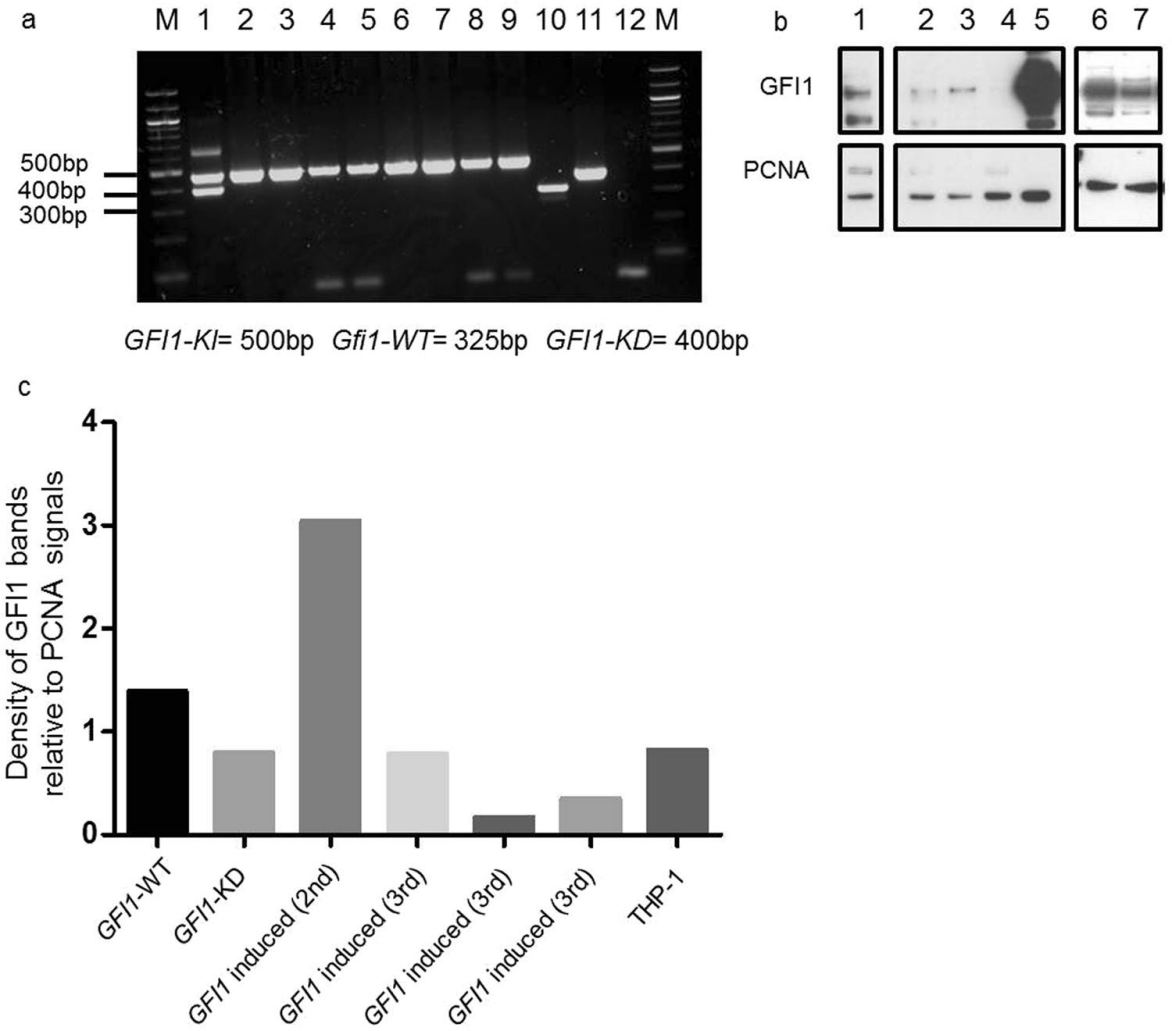
Induced GFI1 upregulation in a humanized GFI1-KD AML mouse model. (**a**) PCR technique as a proof of presence or absence of the Neo cassette and the resulting knock down of *GFI1* expression in different mice. M = Marker; 1 = *GFI1-KDxMx1-Cre*; 2 = *GFI1-KDxMx1-Cre-MLL-AF9* primary transplanted; 3 = *GFI1* induced (2^nd^); 4 = *GFI1* induced (2^nd^); 5 = *GFI1* induced (2^nd^); 6 = *GFI1* induced (2^nd^); 7 = *GFI1* induced (3^rd^), 8 = *GFI1* induced (3^rd^), 9 = *GFI1* induced (3^rd^); 10 *Gfi1*-WT; 11 = *GFI1*-*KD -MLL-AF9*, 12 = negative control. *GFI1-KI* = 500 bp; *GFI1-WT* = 325 bp; *GFI1-KD* = 400 bp. *GFI1* induced = knock down of the *GFI1* gene is reverted and *GFI1* is expressed at normal level. (**b**) Western blot analysis of GFI1 protein levels in BM cells of different mice. 1 = *GFI1* induced (2^nd^); 2 = *GFI1* induced (3^rd^) 3 = *GFI1* induced (3^rd^); 4 = *GFI1* induced (3^rd^); 5 = THP-1, 6 = *Gfi1*-WT; 7 = *GFI1-KD*. *GFI1* induced = knock down of the GFI1 gene is reverted and *GFI1* is expressed at normal level. Presented is the grouping of two different blots. (For full length blots see Supplementary Fig. S7). (**c**) Density of different GFI1 bands in Western Blot relative to PCNA bands. Protein extracts from transgenic and control THP-1 cells were analyzed. 1 = *Gfi1*-WT; 2 = *GFI1-KD*; 3 = *GFI1* induced (2nd); 4 = *GFI1* induced (3rd); 5 = *GFI1* induced (3rd); 6 = *GFI1* induced (3rd); 7 = THP-1. *GFI1* induced = knock down of the *GFI1* gene is reverted and *GFI1* is expressed at normal level. 2nd = secondary recipient mice, 3rd = tertiary recipient mice.

To investigate the effect of increased expression of GFI1 in leukaemia development and to confirm our findings with the AML1-ETOtr model, we used a humanized mouse strain which expresses the human *GFI1* at a reduced level (*GFI1*-KD, KD = knock down)^26^. In this mouse model, the murine *Gfi1* locus was replaced by the human GFI1-encoding cDNA alongside with a Neomycin (*Neo*) selection cassette flanked by loxP sites inserted in antisense direction. The presence of the Neo cassette leads to 5–15% reduced *GFI1* expression compared to the levels expressed in WT mice^26,32^. This level is comparable to the level of *GFI1* in AML patients with reduced *GFI1* expression^26,32^. The removal of the *Neo* cassette results in normal *GFI1* mRNA expression levels. This can be achieved by activation of a Cre recombinase upon intraperitoneal (IP) administration of polyriboinosinic acid/polyribocytidylic acid (poly (I:C)) into *GFI1*-KD mice mated with Mx1-Cre transgenic mice (Fig. 5a). To investigate the effect of increased expression of *GFI1* in leukaemia development and to confirm our findings with the *AML1*-*ETO*tr model, we used an additional model. We retrovirally transduced Lin^−^ BM cells derived from *GFI1*-KD or *GFI1*-KD; *Mx1*-*Cre* transgenic mice with a vector encoding the MLL-AF9 fusion protein, the product of the t(9;11)(p22;q23) translocation, found in a subset of human leukaemia and described to induce an aggressive form of AML in mice, similar to the situation found in human patients^33^. Transplantation of 1 × 10^5^ eGFP^+^ cells (*MLL-AF9*-transduced cells) into WT lethally irradiated mice lead to AML development within about 80 days. The BM leukemic cells arising from these mice were collected and 1 × 10^5^ eGFP^+^ cells were re-transplanted into sublethally irradiated secondary recipient mice. Two days after transplantation, we injected the mice intraperitoneally with poly (I:C) nine times every second day in order to induce removal of the *Neo* cassette, which would result in upregulated expression of *GFI1*. Mice transplanted with *GFI1*-KD cells were also injected with poly (I:C) but since there was no MxCre present, the *Neo* cassette could not be removed (Fig. 5b). Mice transplanted with *GFI1*-KD *MLL-AF9* leukemic cells died as a result of AML development characterized by an increased number of immature myeloid cells (Fig. 5c,d). In contrast, following transplantation with Mx1-CretgGFI1-KD/MLL-AF9 leukemic cells and after poly (I:C) treatment, the GFI1 level was upregulated (Fig. 5e) leading to increased GFI1 expression. We observed that the leukemic cells show upregulation of myeloid markers as demonstrated by staining for myeloid markers and in addition a change in morphological appearance, as seen in the cytospin preparations (Fig. 5c,d,f). In addition, we observed a reduction of number of cKit^+^ cells (Supplementary Fig. S4e). CKit^+^ cells represent a fraction, which is enriched for leukemic stem cells in MLL-AF9 associated AML^27^. Finally, we observed a significant increase in the rate of apoptosis (Fig. 5g). To prove the functionality of the poly (I:C) treatment and the activation of the Mx1-Cre recombinase we generated a PCR assay, demonstrating that the *Neo* cassette was successfully cut out and the *GFI1*-KD phenotype was reverted (Fig. 6a). Furthermore, the upregulation of the GFI1 protein level could be shown by Western blot in the secondary transplanted mice (Fig. 6b,c). We could not observe an increase in survival or significant changes in pathological parameters like spleen size or organ infiltration in mice over-expressing *GFI1* but observed a clear reduction of cKit^+^ cells, which is a fraction of cells, in which leukemic stem cells are enriched^27^, giving a first hint that upregulation of GFI1 also alters *in vivo* the characteristics of leukaemia (Supplementary Fig. S4).

We finally aimed to investigate whether upregulation of *GFI1* affects the ability of the leukemic cells to generate a transplantable leukaemia. To this end we transplanted leukemic cells with upregulated expression of *GFI1* into tertiary mice to determine the number of leukemic stem cells using a limiting dilution assay (Fig. 5b). Upon disease development we analysed the mice with supposedly upregulated expression of *GFI1*. To our surprise, the clear differentiation toward granulocytes was neither observed by cytologic examination nor by immunophenotyping (Supplementary Figs S5 and S6). Using PCR, we could still detect the removal of the *Neo* cassette (Fig. 6a), however by Western blot analysis, the upregulated levels of GFI1 observed in the transplanted cells were not observed any more in the cells emerging after tertiary transplantation (Fig. 6b,c and Supplementary Fig. S7). Despite persisting removal of the *Neo* cassette, the emerging, tertiary transplanted leukemic cells with removed *Neo* cassette have found ways to reduce GFI1 levels. Due to scarcity of material and limitations by federal law to perform mouse experiments, we were not able to determine the exact mechanism.

Taken together, all of these results suggest that upregulation of *GFI1* gene expression in AML cell lines and mouse models of AML inhibits the *in vitro* cell proliferation and leads to changes of morphological and functional characteristics of leukemic cells *in vivo*, indicating that GFI1 levels play a critical role in leukaemia progression.

## Discussion

A recent study described that Gfi1 loss of function induced the progression of myeloproliferative disorder (MPD) to AML in a K-ras mouse model^22^. In *de novo* chronic phase-chronic myeloid leukaemia (CP–CML) patients, low *GFI1* expression was associated with an increased risk of transformation to blast crisis^34^. In addition, downregulation of *GFI1* expression in CD34^+^ cells derived from CML patients resulted in significantly more clonogenic cells^35^. In contrast, ectopic expression of *GFI1* in *BCR-ABL*-expressing cells inhibited their proliferation and colony formation capacity^35^. Furthermore, forced expression of *Gfi1* in murine Lin^−^ BM cells extinguished their replating capacity^22^. Recently, we reported that reduced levels of GFI1 were associated with an inferior prognosis of AML patients^26^. Based on these observations, we hypothesized that *GFI1* overexpression may suppress AML cell growth.

In this study we demonstrate that upregulated expression of *GFI1* in human UCB-derived HSPCs decreased the frequency of LMPPs and increased the fraction of EMPs, showing that upregulated expression of *GFI1* drives cells into differentiation. Moreover, in several widely used human AML cell lines, *GFI1* overexpression inhibited cell growth and colony formation, while promoting differentiation of cells. Consistent with these findings, the co-expression of the truncated forms of AML1-ETO and GFI1 in murine Lin^−^ BM cells impeded their replating capacity, GFP^+^ cells being counter-selected over time. The relative m-RNA level between empty vector-transduced and *GFI1*-expressing vector-transduced cells ranged between 5–200 fold, but was similar to the *GFI1* m-RNA level between low *GFI1*-RNA expressing AML cell lines (such as KG-1 and K-562) and *GFI1*- high expressing cell lines (such as Kasumi-1 and THP-1), thus experimentally induced *GFI1* RNA-levels were within range compared to physiological levels. Despite the high differences at the RNA level, the protein level found in the cell lines were either at the same level or increased 2-3 fold compared to Kasumi-1, THP-1, or primary cells, thus the effects are not due to very high ectopic expression. We finally studied the effect of elevated GFI1 levels on MLL-AF9-induced leukaemogenesis *in vivo*. Using a humanized *GFI1*-KD mouse model, which expresses *GFI1* at a similar level as found in certain subgroups of human AML^26^. We show that upregulation of *GFI1* expression *in vivo* promotes differentiation and apoptosis of the leukemic cells and reduces the number of cKit^+^ cells, which is a fraction enriched for leukemic stem cells^27^. We then investigated whether upregulated expression of *GFI1* impedes the ability of cells to reconstitute leukaemia after transplantation. To this end, we tertiary transplanted these *GFI1* KD *Mx1*-*Cre* tg leukemic cells with upregulated expression of *GFI1*. When then examined the GFI1 protein level in the emerging leukemic cells. To our surprise only leukemic cells, which found means to reduce *GFI1* expression despite removal of the *Neo* cassette, emerged, or at least these cells competed better against leukemic cells with increased level of expression.  The difference in protein level between low and elevated expression in the different secondary and tertiary transplanted cells varied only in a range of 2-3 fold, pointing to the hypothesis that leukemic cells depend on a tightly controlled reduced expression of *GFI1* to strive *in vitro* and *in vivo*.

Our findings reveal that an increase of the GFI1 level in leukemic cells impedes the expansion of leukemic cells *in vitro*. *In vivo*, upregulation of GFI1 leads to differentiation of leukemic cells both on a morphological level and with regard to expression of myeloid markers. We previously demonstrated that a reduced level of GFI1 promotes AML development and complete loss of Gfi1 impedes development of lymphoid leukaemia^26,36^. These data, together with our data indicate that GFI1 plays a dose-dependent role in leukaemia. Although more work is needed to examine specifically the role of reduced levels in lymphoid leukaemia or loss of Gfi1 in myeloid leukaemia, we postulate that a certain low degree of *Gfi1* expression is beneficial for myeloid and lymphoid leukaemia and that deviation from this level disturbs leukaemia progression and would represent an approach for therapeutic intervention. In summary, this study demonstrates, as a proof of concept, that upregulated expression of *GFI1* is a valid approach to target leukemic cells. Previous work and our own data has shown, that overexpression of *GFI1* does not interfere with the viability of the haematopoietic system and hence targeting *GFI1* levels could be a gateway to a novel therapy^37^. Future work has to examine whether a specific GFI1 degrading pathway or signalling cascades can be targeted to enhance activity of GFI1 as current data hint to this point^38^.

## Materials and Methods

### Cell preparation and cell culture

Human Umbilical Cord Blood (UCB) cells were obtained from unrelated donors after informed consent from all subjects according to the Declaration of Helsinki. All experiments with human samples were carried out in accordance with the approved protocol of the University of Duisburg-Essen ethics committee (ethikkommission@uk-essen.de), who approved on 28.08.2014 all studies with human samples under the document number 13-5675-BO. All methods were carried out in accordance with relevant guidelines and regulations. Informed consent was obtained from all subjects. Mononuclear cells (MNCs) were isolated from individual sources by Ficoll (Biocoll Separating Solution, Biochrom AG, Berlin, Germany) density gradient centrifugation and CD34^+^ cells were highly enriched by magnetic cell separation as described previously^39^. Cells were cultured in a humidified atmosphere at 37 °C and 5% CO_2_ at a density of 1 × 10^5^ cells/mL in IMDM medium (Lonza, Cologne, Germany) supplemented with 20% FBS (Biochrom, Berlin, Germany), 100 U/mL penicillin and 100 U/mL streptomycin (Life Technologies, Darmstadt, Germany) and with the following cytokines: human Flt3-Ligand, human SCF and human TPO, each at 10 ng/mL final concentration (all from PeproTech, Rocky Hill, NJ, USA).

THP-1 cells were maintained in RPMI1640 medium (Lonza, Cologne, Germany) supplemented with 10% FBS (Biochrom, Berlin, Germany), 100 U/mL penicillin and 100 U/mL streptomycin (Life Technologies, Darmstadt, Germany). KG-1 and Kasumi-1 cells were maintained in RPMI1640 medium (Lonza, Cologne, Germany) supplemented with 20% FBS (Biochrom, Berlin, Germany), 100 U/mL penicillin and 100 U/mL streptomycin (Life Technologies, Darmstadt, Germany). K-562 cells were maintained in IMDM medium (Thermo Fisher Scientific, Dreieich, Germany) supplemented with 10% FBS (PAN Biotech GmbH, Aidenbach, Germany), and 1% penicillin-streptomycin (Sigma-Aldrich Chemie GmbH, Munich, Germany).

Murine BM cells were isolated from femurs, tibiae and humeri of 8–12 week old mice. Magnetic separation of Lineage negative (Lin^−^) BM cells was performed using a Lineage Cell Depletion Kit, mouse (MiltenyiBiotec, BergischGladbach, Germany) according to manufacturer’s instructions. For *in vitro* studies, Lin^−^ cells were cultured in IMDM medium (Thermo Fisher Scientific, Dreieich, Germany) supplemented with 20% FBS (PAN Biotech GmbH, Aidenbach, Germany), 1% penicillin-streptomycin (Sigma-Aldrich Chemie GmbH, Munich, Germany) and the following cytokines: 20 ng/ml recombinant mouse Stem Cell Factor (SCF), 10 ng/ml recombinant mouse Interleukin-3 (IL-3) and 10 ng/ml recombinant human Interleukin-6 (IL-6) (all from MiltenyiBiotec, Bergisch Gladbach, Germany). For *in vivo* studies, Lin^−^ cells were cultured in Marrow Max Bone Marrow Medium (Thermo Fisher Scientific, Dreieich, Germany) supplemented with 20% FBS (PAN Biotech GmbH), 1% penicillin-streptomycin (Sigma) and the same cytokines in the same concentrations mentioned above.

### Lentiviral and retroviral transduction

Lentiviral supernatants were generated as described previously^40^. Briefly, HEK293T cells were co-transfected with either IRES-eGFP (control) or GFI1-IRES-eGFP (GFI1-eGFP)-encoding plasmids (pCL6-IRES-EGwo backbone)^41^, the pCD/NL-BH helper plasmid^42^ and the codon-optimized, human foamy virus envelope expression plasmid pcoPE^43^, using Jetpei (Polyplus, IllkirchCedex, France) transfection reagent according to the manufacturer’s recommendations. Gene expression from the human cytomegalovirus (CMV) immediate-early gene enhancer/promoter was induced 24 hours after transfection with 10 mM sodium butyrate. 48 hours after transfection, supernatants were collected, filtered through 0.45 μM filters (Sartorius), concentrated by centrifugation at 25,000 × g for 90 min at 4 °C and stored at −80 °C. Virus stocks were titrated on HEK293T cells before use.

A number of 5 × 10^4^ target cells (CD34^+^, KG-1, THP-1, Kasumi-1 and K-562) were co-cultured overnight with lentivirus stocks for transduction and overexpression of the eGFP and GFI1-eGFP, individually. After incubation with virus, cells were washed with PBS and transferred to a new 24 well plate. KG-1, THP-1, Kasumi-1 and K-562 cells were passaged at least twice after transduction and subsequently eGFP^+^ cells were sort-purified on a BD FACS Aria IIIu cell sorter.

Retroviral supernatants were produced by calcium phosphate co-transfection of 293Tmyc cells with 2.25 μg pCL-Eco retroviral packaging vector (IMGENEX, San Diego, USA) and 20 µg of either of the following retroviral plasmid DNA: pMig-AEtr-i-tdTOM (shortly, AML1-ETOtr-TOM, kindly provided by Dr. Michael Grez and Dr. Christian Wichmann), MCSV-MLL-AF9-IRES-GFP (shortly MLL-AF9, generously provided by Dr. Jay Hess), eGFP and GFI1-eGFP. At 48 and 72 hours post-transfection the virus supernatants were collected, filtered through 0.45 μM filters, and stored at −80 °C. The viral supernatants were titrated on murine NIH3T3 cells. Retroviral transduction of Lin^−^ BM cells was done on days three and four after isolation. Viral supernatants were centrifuged twice onto RetroNectin-coated (Takara Bio Inc., Nojihigashi, Japan) 24 well non-tissue culture treated plates at 4600 rpm for at least 90 minutes at 4 °C. Upon removal of most of the viral supernatants from the retronectin-coated wells, 1 × 10^6^ Lin^−^ cells were added to each well. The plates were centrifuged for 10 min at 1200 rpm at 4 °C and 2 µg/ml of polybrene infection/transfection reagent (Merck KGaA, Darmstadt, Germany) was added to each well.

### Mouse strains

Generation of the GFI1-KD mouse model was previously described^26^. Mx1-Cre mice were purchased form Jackson Laboratory (Bar Harbor, ME, USA). C57BL/6 J mice were bred in the animal facility of University Hospital Essen. All mice were housed in single ventilated cages and specific pathogen-free conditions in the animal facility of University Hospital Essen. All experiments on animals were carried out in accordance with the approved protocol of the government ethics committee for animal use, who approved on 21.07.2011 all studies on animals under the document number G1196/11 (fachbereich84@lanuv.nrw.de).

### Transplantation of murine leukemic or pre-leukemic hematopoietic progenitors

On day 5 after isolation, 1 × 10^5^ GFP-positive MLL-AF9-expressing cells derived from GFI1-KD or GFI1-KD; Mx1-Cre transgenic mice were injected intravenously (IV) into lethally irradiated (10 Gy) WT recipient mice together with 1.5 × 10^5^ competitive BM cells. Mice were regularly monitored for any signs of leukemia development. Once moribund, the mice were sacrificed and analyzed. For secondary transplantations, 1 × 10^5^ GFP^+^ GFI1-KD and GFI1-KD;Mx1-Cre BM leukemic cells were injected IV into sublethally irradiated (3 Gy) WT recipient mice. Poly (I:C) was administered intraperitoneally (IP) starting two days after transplantation every second day for a total of 9 times. Once moribund, the mice were sacrificed and analyzed. For tertiary transplantations, 5 × 10^4^ GFP^+^ BM leukemic cells were injected into sublethally irradiated WT mice. Poly (I:C) was not administered into tertiary recipient mice. Once the mice showed signed of leukemia, the mice were sacrificed and analyzed.

### Poly (I:C) treatment

Mx1-Cre mice that harbor the poly (I:C) inducible Cre recombinase gene under the control of Mx1 promoter were crossed to GFI1-KD mice, in which the murine Gfi1 locus was replaced by the human GFI1-encoding cDNA alongside with a Neomycin (Neo) selection cassette flanked by loxP sites inserted in antisense direction. To remove the Neo cassette, whose presence leads to a reduced GFI1 expression of about 5–15% of the levels expressed in WT mice, mice that were previously transplanted with MLL-AF9 leukemic cells derived either from GFI1-KD or GFI1-KD; Mx1-Cre transgenic mice were injected intraperitoneally (IP) nine times every second day with polyriboinosinic acid/polyribocytidylic acid poly (I:C) (Sigma-Aldrich Chemie GmbH, Munich, Germany).

### Flow cytometric analysis

After 6 days in culture transduced CD34^+^ cells were harvested and stained with the following fluorochrome conjugated antibodies: anti-CD34-APC-AF750, clone 581 (Beckman Coulter, California, United States), anti-CD133-PE, clone AC133 (MiltenyiBiotec, BergischGladbach, Germany), anti-CD45RA-PE-Cy7, clone HI100 (BioLegend, London, United Kingdom), anti-CD38-PerCPCy5.5, clone HIT2 (BD Biosciences) and anti-CD10-PeCF594, clone HI10a (BD Biosciences, Heidelberg, Germany). Dead cells were excluded by DAPI staining. CD34^+^CD133^+^CD45RA^+^ HSPCs were defined as LMPP-enriched fraction and CD34^+^CD133^low^CD45RA^−^ HSPCs as EMP-enriched fraction. The measurements were performed in a BD FACSAriaIIIu (BD Biosciences, Heidelberg, Germany) flow cytometer. Unmanipulated cells and eGFP overexpressing cells were used as experimental controls. The data was analyzed using FlowJo7.6.5. Software.

Bone marrow and spleen cells from leukemic mice were analyzed by flow cytometry (FACS) using the following anti-mouse antibodies from Biolegend (London, United Kingdom): PE anti-mouse Ly-6G/Ly-6C (Gr-1), clone RB6-8C5, PerCP/Cy5.5 anti-mouse/human CD11b, clone M1/70, PE anti-mouse CD8a, clone 53-6.7,PerCP/Cy5.5 anti-mouse CD4, clone GK1.5, PE anti-mouse TER-119/Erythroid Cells, clone TER119, PerCP anti mouse/human CD45R/B220, clone RA3-6B2 and APC anti-mouse CD117(c-Kit), clone 2B8. Apoptosis was measured by staining the cells with Annexin V (BD Bioscience or Biolegend).

### CFC assay

CFC assays with human cells were performed as described previously^29^. Briefly, 100 THP-1 and KG-1 sort-purified cells (eGFP^+^) were seeded into Methocult GF H4434 medium (Stemcell Technologies Vancouver, Canada). Colonies were observed via microscopy after 14 days. Cells were harvested from CFC assays for cytospin preparation as described previously^29^.

For CFC assays with murine cells, approximately 1000 sorted GFP^+^ cells were seeded in methylcellulose (Stemcell Technologies, M3434). The number of colonies as well as the cell number was assessed 7 to 10 days later.

### RNA isolation, cDNA synthesis and qRT-PCR

Total RNA was extracted from human primary cells, the human AML cell lines and bone marrow cells of leukemic mice using the RNeasy MiniKit (Qiagen, Hilden, Germany) according to the manufacturer’s instructions. The concentration and purity of the total RNA was determined with a Nanometer P-class USB micro photometer (Implen, Munich, Germany). cDNA synthesis was performed using A Clontech Laboratories PCR kit (Takara, Kyoto, Japan) according to the manufacturer’s instructions. qRT-PCR was performed in the Real Time PCR system OneStep (Applied Biosystems, Thermo Fisher Scientific, Schwerte, Germany). The following TaqMan primers (Applied Biosystems) were used: human GFI1 (Hs01090305_m1) and human GAPDH (Hs04420697_g1).

### Western Blot

Primary human CD34^+^ cells, AML cell lines transduced with eGFP and GFI1-eGFP lentiviral vectors were sorted and nuclear proteins were extracted using the NE-PER Nuclear and Cytoplasmic Extraction Reagents (Thermo Scientific, Darmstadt, Germany). Antibodies used for Western blotting included goat polyclonal antibody against GFI1 (N-20) (1:1000, G6670, Sigma-Aldrich, Darmstadt, Germany), rabbit polyclonal antibody against PCNA (1:500, sc-7907, Santa Cruz Biotechnologies, Minneapolis, USA), donkey anti-goat IgG-HRP (1:5000, sc-2020, Santa Cruz Biotechnologies) and Goat anti-rabbit IgG-HRP (1:2500, sc-2030, Santa Cruz Biotechnologies). ECL Western blot analysis system (Amersham) was used for detection.

### PCR

To proof if the Neo cassette was entirely cut out, we performed a PCR like previously described^31^.

## Electronic supplementary material

Supplementary information
