# Supplementary material for: Enforced GFI1 expression impedes human and murine leukemic cell growth
Source: Sci Rep. 2017 Nov 16;7:15720. doi: 10.1038/s41598-017-15866-9 (PMC5691148; doi:10.1038/s41598-017-15866-9)
Supplement: Supplementary file 1 — Supplementary information [file 41598_2017_15866_MOESM1_ESM.pdf]

Supplementary

Enforced GFI1 expression impedes human and murine leukaemic cell growth

Judith M. Hönes[1,4#], Aniththa Thivakaran[1#], Lacramioara Botezatu[1#], Pradeep Patnana[1], Symone Vitoriano da Conceição Castro[2,3], Yahya S. Al-Matary[1], Judith Schütte[1], Karen B. I. Fischer[1], Lothar Vassen[1], André Görgens[2,5], Ulrich Dührsen[1], Bernd Giebel[2], and Cyrus Khandanpour[1+]

Figure S1:

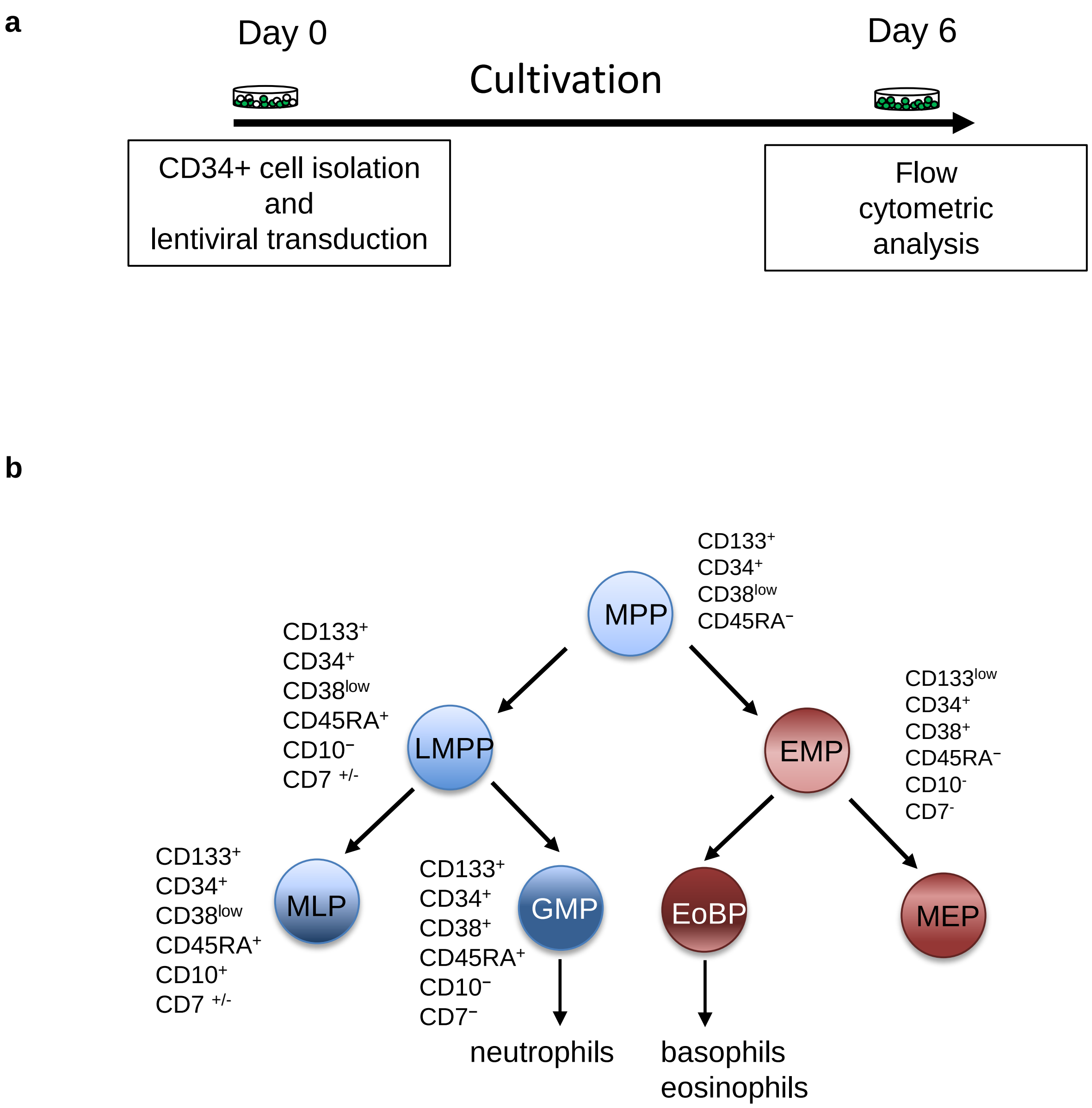

S1: a) Schematic representation of lentiviral transduction of human UCB-derived CD34^{+} cells. After 6 days in culture, transduced CD34^{+} cells were analyzed by multi-color flow cytometry to detect various HSPC populations. b) Model of surface markers from different hematopoietic cells (modified after Görgens et al. 2013).

Figure S2:

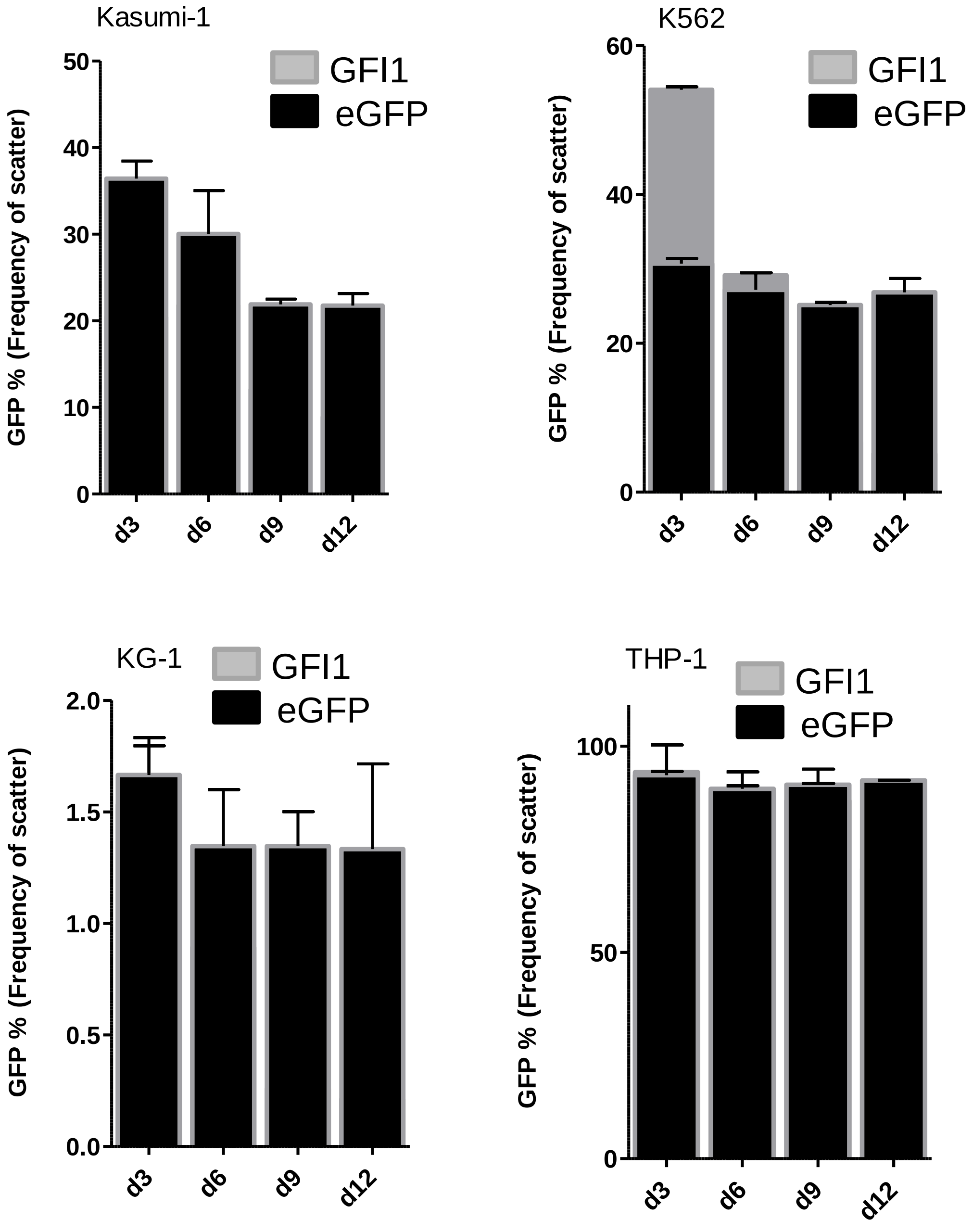

S2: The cells of the different cell lines were transduced on day 0 and kept in culture for a total of 12 days. On days 3, 6, 9 and 12 an aliquot of the cells was measured by FACS for checking the eGFP frequency.

Figure S3:

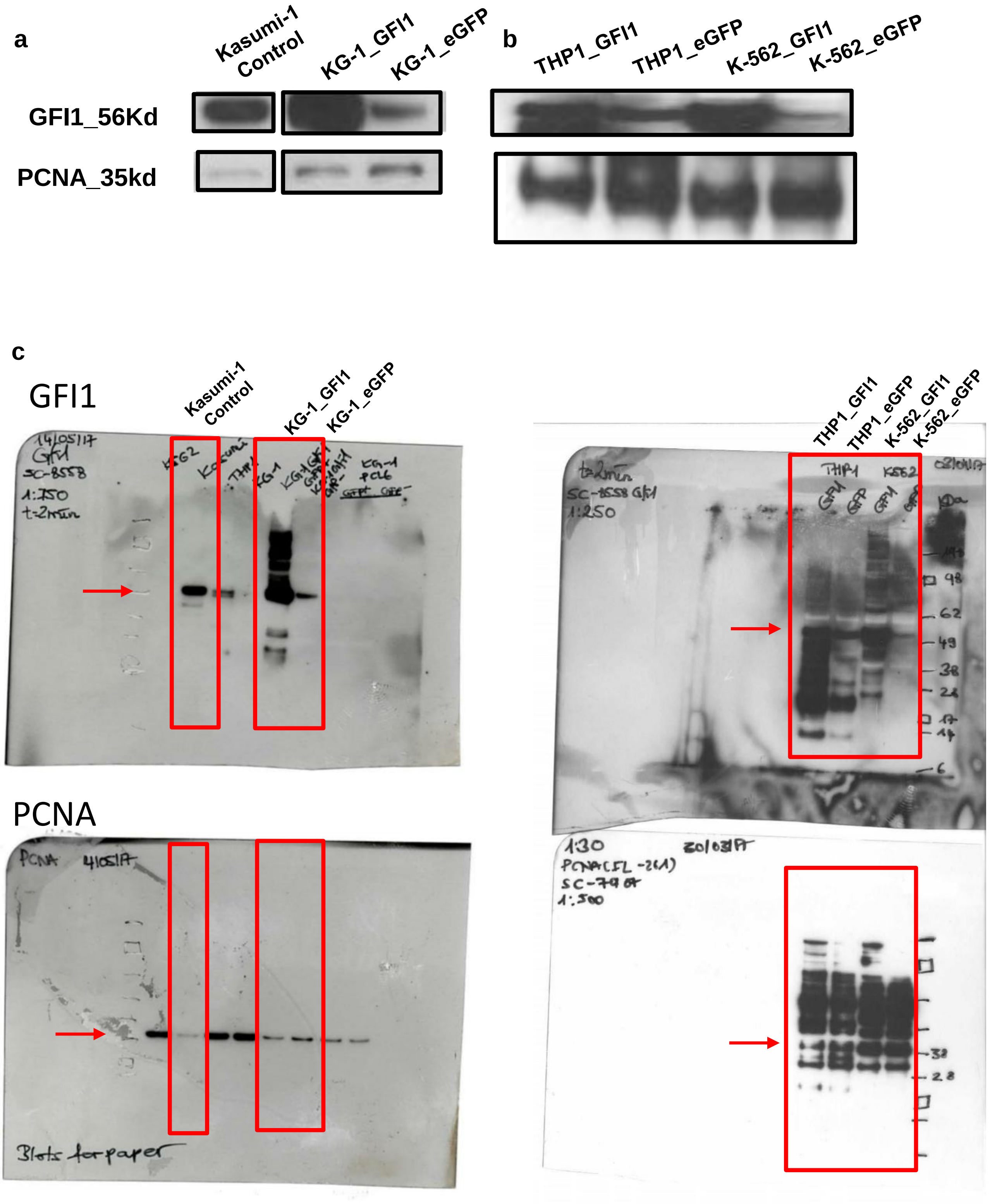

S3: a) Western blot analysis of GFI1 in Kasumi-1 and KG-1 cells transduced either with *eGFP* or *GFI1* lentiviral vectors. Grouping of blots cropped from the same blot. b) Western blot analysis of GFI1 in THP-1 and K-562 cells transduced either with *eGFP* or *GFI1* lentiviral vectors. c) Full length Western blots of the cropped figures S3a and S3b. Arrows mark the appropiate protein band.

Figure S4:

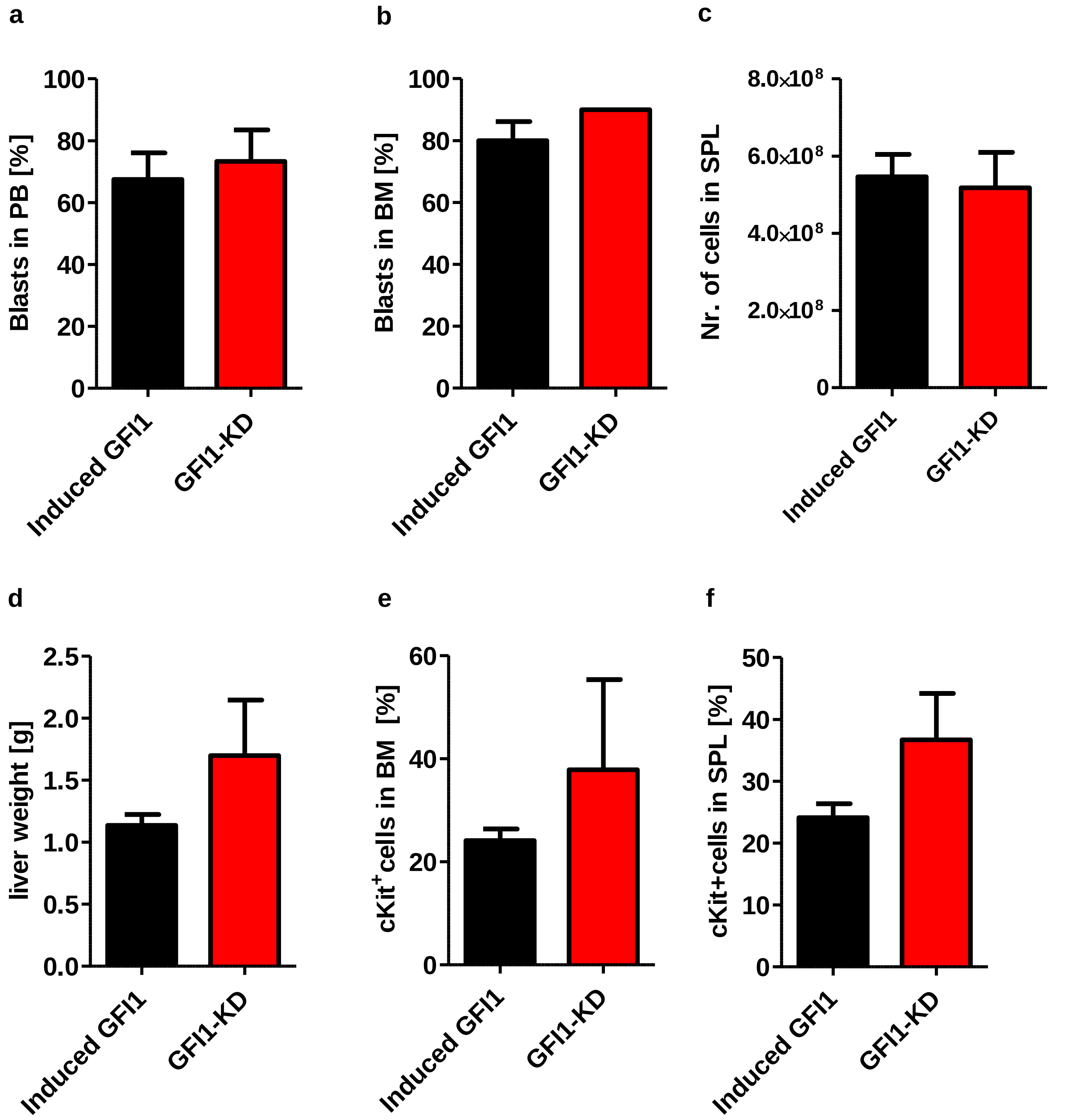

S4: a) Percentage of blasts in peripheral blood of secondary transplanted *MLL-AF9* mice (n=4). b) Percentage of blasts in BM of secondary transplanted *MLL-AF9* mice (n=4). c) Number of cells in SPL from secondary transplanted *MLL-AF9* mice (n=4). d) Liver weight of secondary transplanted *MLL-AF9* mice (n=4). e) Percentage of cKit positive cells in BM from secondary transplanted *MLL-AF9* mice (n=4). f) Percentage of cKit positive cells in SPL from secondary transplanted *MLL-AF9* mice (n=4).

Figure S5:

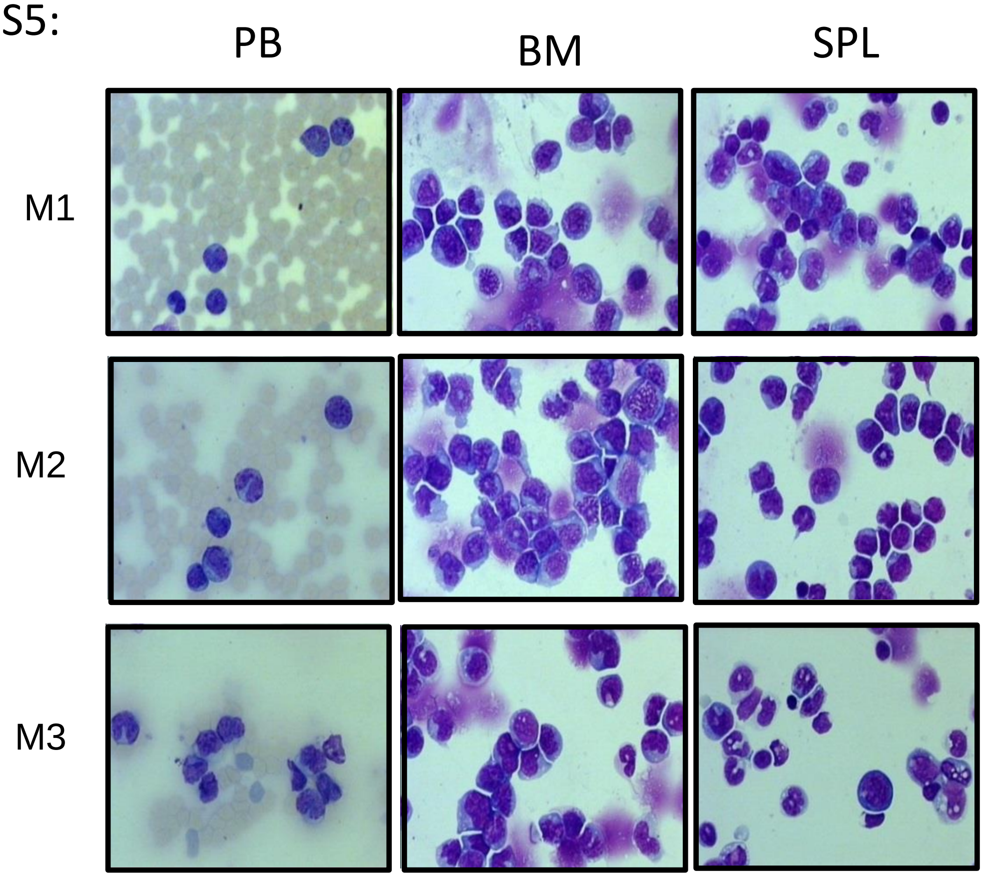

S5: Pictures of blood smears and cytospins of BM and SPL from tertiary transplanted mice that expressed *GFI1-MLL-AF9* bone marrow cells. M1= mouse1, M2= mouse 2, M3= mouse 3.

Figure S6:

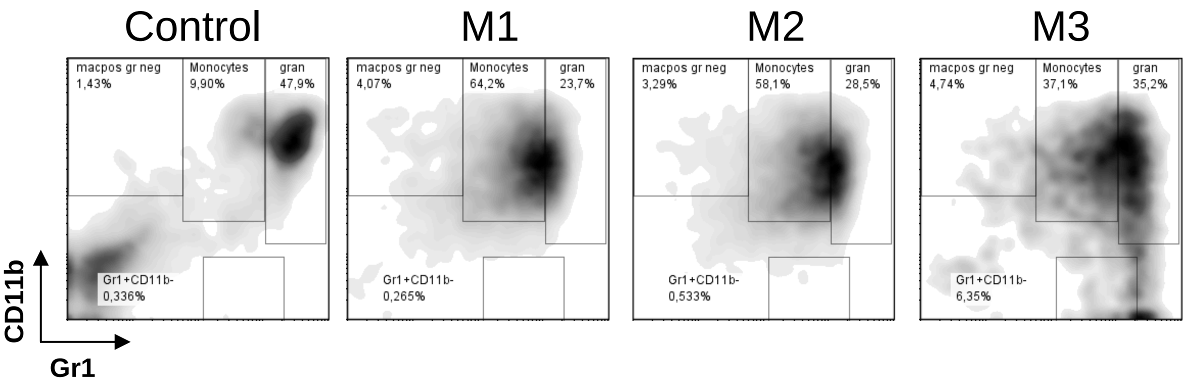

S6: Flow cytometric analysis of CD11b and Gr-1 positive BMcells from three different tertiary transplanted mice with *GFI1* induced *MLL-AF9* bone marrow cells. M1= mouse1, M2= mouse 2, M3= mouse 3.

Figure S7:

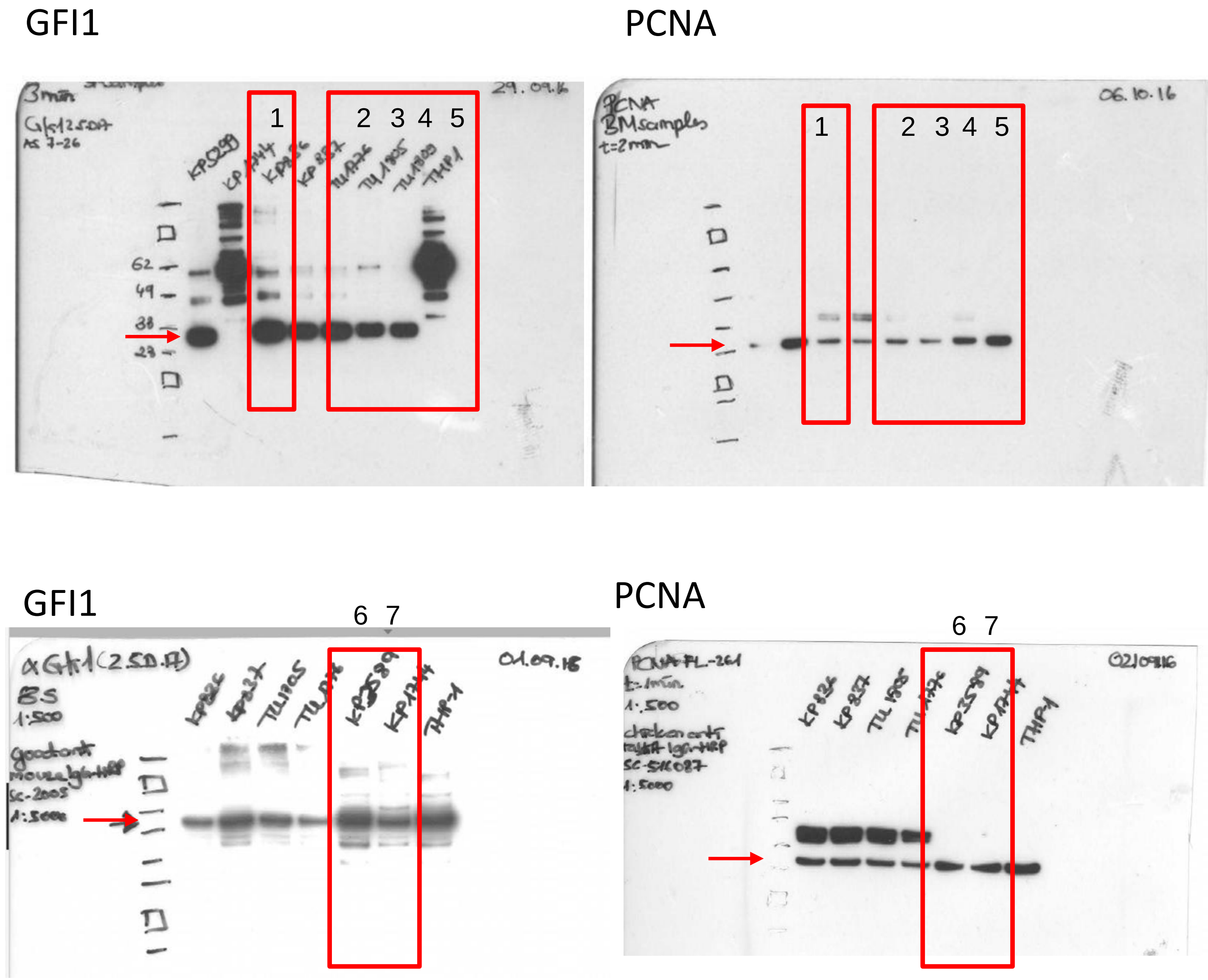

S7: Full length Western blots. Here are presented the full length Western Blots from the cropped blots shown in the main Figure 6b. The used lanes are marked with the red box. 1= GFI1 induced (2nd); 2= GFI1 induced (3rd) 3= GFI1 induced (3rd); 4= GFI1 induced (3rd); 5= THP-1, 6= Gfi1-WT; 7= GFI1-KD. Arrows mark the appropiate protein band.
